# Balanced assessment systems revisited

**DOI:** 10.1080/20016689.2017.1355190

**Published:** 2017-07-26

**Authors:** Dávid Dankó, Márk Péter Molnár

**Affiliations:** ^a^ Institute of Management, Corvinus University of Budapest, Budapest, Hungary; ^b^ Ideas & Solutions, Budapest, Hungary

**Keywords:** Health technology assessment (HTA), balanced assessment, pragmatic HTA, MCDA, emerging markets, value assessment

## Abstract

In 2014, balanced assessment systems (BAS) were proposed as a resource-conscious, ‘fit-for-purpose’ form of health technology assessment for middle-income countries which lack resources and competences necessary for resource-intensive health technology assessment models. BAS has undergone extensive policy debate in the period since its publication but it has not been critically assessed in a structured form yet. This article aims to describe both the contributions and the weak spots of the original framework and to reflect on them with the intention of further developing the model.

## Introduction

**Figure 1.**
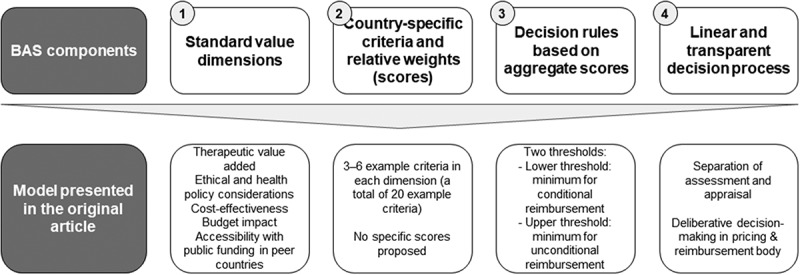
Logic of a balanced assessment system [1].

The original idea of balanced assessment systems (BAS) was proposed in an article published in *JMAHP* in early 2014 [1] as a ‘fit-for-purpose’ form of multi-criteria health technology assessment (HTA) for middle-income countries which lack resources and competences necessary for more resource-intensive HTA frameworks such as cost-effectiveness analyses or comparative benefit assessment. The basic idea of BAS is that it is possible to define standard economic, clinical and other value dimensions in which system-specific criteria can be defined and used, with a scoring algorithm, for the prioritization of new health technologies, especially medicines (Figure 1).

In the past three years, BAS has received considerable interest: it has served as the basis for HTA system design proposals and/or has been discussed in policy debates and workshops in several Central European countries, most prominently in Serbia, Bulgaria and Slovakia [2–4] where it is been referred to as ‘pragmatic balanced assessment (PBA)’. A form of BAS, ‘structured quick assessment’, has been incorporated into the pharmacoeconomic guidelines of India [5]. Furthermore, BAS has also been discussed at health policy events in Middle Eastern countries (see Dankó [6]) and in Russia, a country currently in the process of building its HTA system [7,8].

Although originally proposed from a payer’s perspective and for middle-income countries, BAS has gained attention from non-governmental stakeholders such as patient advocacy groups and pharmaceutical industry associations (see EFPIA HTA Group [4]) in high-income countries as well. The appeal of balanced assessment towards these stakeholders has been its pragmatic focus (i.e., concrete applicability) on informing policy and decision-making, its goal to establish a direct link between health technology assessment, pricing and (de)listing decisions, and that BAS advocates the explicit recognition of added clinical benefit in HTA processes the way it is institutionalized practice in comparative assessment systems, thus shifting away from budget-focused decision-making.

Awareness and acceptance of balanced assessment by the scientific HTA community itself have remained limited so far, although it has been presented as an emerging alternative value framework in the HTAi Policy Forum 2017 Background Paper [9]. Presentations and roundtable sessions about pragmatic HTA in general (and the BAS model in particular) have evoked mixed reactions – both positive and critical comments – from HTA scholars at major HTA conferences (e.g., ISPOR Amsterdam in 2014, HTAi Oslo in 2015, HTAi Tokyo in 2016). In some emerging pharmaceutical markets, balanced assessment has met resistance from HTA scientific leaders who may have misunderstood it as a threat to academic achievements previously reached within the HTA community, and from some budget holders who expressed concerns that the formulaic decision rules of the BAS model might reduce their ability to control their budgets.

In retrospect, the original article seems to have provided a comprehensive critical overview of the key elements of institutional environments in middle-income (non-core) pharmaceutical markets, and gave a plausible analysis of why economic evaluation may fail and qualitative/comparative assessment may be insufficient in these countries. The viability of the framework proposed by the article has also been proven by pilot assessment. Being a first version, however, the model published in 2014 is not void of conceptual weaknesses, as policy dialogue since its publication has revealed. This article therefore aims at taking a self-critical look at BAS by recapitulating its contributions to public debate on HTA and by addressing both the shortcomings of the original framework and possible answers to overcome these. Through these possible answers, we also intend to show some future perspectives for pragmatic alternative value frameworks.

## Contributions of balanced assessment systems to the public debate on HTA

The balanced assessment model has contributed to the public debate on HTA in at least four major aspects:

- *Role of the institutional environment*. Firstly, BAS has pointed out that many countries in the process of designing and implementing health technology assessment systems do not have either resources or capabilities (or both) to consider fully fledged HTA models for implementation (see the HTA Core Model® for a description of a comprehensive HTA framework [10]), and that existing resources and capabilities must be regarded as crucial contextual variables. By emphasizing the importance of resources and capabilities, the BAS model seems to have drawn some attention to the role of the institutional environment in the field of HTA. It has also revealed some shortcomings of positivist-absolutist-normative approaches to HTA and corroborated that there is no one best way to building health technology assessment systems. Instead, the proper choice is always context-dependent.
- *Link to health policy*. Secondly, and equally importantly, BAS advocates that as HTA informs complex decision processes which include lay and political stakeholders and influencers, it should be approached and discussed from a policy perspective. HTA should be steered mainly by the purpose of serving policy rather than disseminating science. This manifestly value-laden ‘propensity’ of balanced assessment towards policy-driven HTA (as opposed to methods-driven HTA) has been a recurrent element in policy debate on HTA to the extent that BAS has even been labelled as ‘anti-elitist’ by supporters and ‘destructive’ by opponents at conferences.
- *Multi-criteria assessment*. BAS was proposed at a time when scientific interest in multi-criteria decision analysis (MCDA) [11,12] started to increase considerably. BAS has sometimes indeed been discussed as a pragmatic form of multi-criteria technology assessment inasmuch as it uses multiple decision criteria in multiple pre-defined value dimensions and allocates relative weights (scores) to individual criteria. Moreover, BAS models proposed so far have tried to ensure that drivers of clinical value and drivers of economic value are both considered in pricing and listing processes and a structured approach is used to capture all possible drivers of value for a new medicine. At the same time, the fact that BAS proposes that a universal set of country-specific criteria with standard weights (scores) can be established for decision-making in any given country makes a clear difference from more resource-intensive and scientifically-geared MCDA frameworks.
- *Transferability of assessment results*. As is the case with the generalizability of clinical trial outcomes to broader populations, the transferability of assessment results between systems is a hot topic in HTA [13,14]. The logic of balanced assessment has also highlighted the contradiction between the pragmatic need for using external references (e.g., previous assessment reports or funding decisions) and the legitimate academic and methodological concerns regarding transferability of evidence. Nonetheless it has also become evident that balanced assessment can provide a practical ‘shell’ for embedding future results from the application of the EUnetHTA framework for Rapid Relative Effectiveness Assessment [15].

## Criticism of the original balanced assessment model and possible answers

As mentioned in the Introduction, the original BAS model could not always provide fully mature and comprehensive answers to all the main challenges it set out to address, namely matching assessment methods to the needs of decision-makers and the local institutional context, counterbalancing financially-driven decision patterns through the recognition of clinical, social and ethical sources of value and offering a linear and transparent decision process. Based on the critique accumulated in the past three years, it is possible to draw up a list of its weak spots and to provide solutions or improvements to these. In doing this, we will start with more conceptual questions and head towards more technical considerations.

### HTA paradigms

Upon classifying international HTA systems into three paradigms (economic evaluation, qualitative/comparative assessment, balanced assessment), the original paper suggested balanced assessment as a separate third paradigm. In retrospect, there seems to be some value bias here: as opposed to the other two paradigms which can be characterized by widespread practical application globally, balanced assessment is rather an ideal type. Experts have pointed out that no HTA systems in any real country can be called ‘balanced’ in a way which is entirely indisputable. Australia and Canada, which were used in the original article as proxies, do use non-quantitative criteria and well-established deliberative processes, yet they could well be classified as ‘economic evaluation countries’. The system in Sweden is also based on economic analysis but from a broader societal perspective. On the other hand, qualitative/comparative countries such as France or Italy increasingly require economic evidence for new technologies. Although this might lead to the superficial conclusion that balanced assessment is what is coming up in the form of MCDA, the form MCDA is taking within the scientific HTA community [12,16] is somewhat distant from the objectives of balanced assessment both in terms of pragmatism and the application of pre-defined decision rules based on which clinical, social, ethical and economic sources of value can qualify a medicine for public funding.

This means that while the label ‘balanced assessment’ does not need to be discarded, it must be pointed out that no international system fully corresponds to the ‘ideal type’ definition of balanced assessment. Rather, different systems show different extents and mechanisms for balancing qualitative and quantitative criteria. This is important because it explains why some have found it difficult to put the actual Australian or Canadian system, MCDA and BAS in the same box.

When the original article was published in early 2014, the term ‘value frameworks’ was not as widely known and applied in the HTA domain as today although it had already been introduced into the vocabulary of HTA (see Ontario Citizens Council [17]). The 2013 HTAi Policy Forum background paper [18] still referred to ‘approaches’ and ‘system perspectives’. Now, against a backdrop of rapidly evolving dialogue about value frameworks (and this term getting more and more widespread use), the most suitable approach may be to understand and position balanced assessment as a specific value framework within the much more abstract ‘balanced’ paradigm. In this regard, an alternative denomination for the model, PBA, may even be more appropriate.

### Decision rules

The original BAS model permits several decision rules but highlights only one: a scoring framework with two cut-off points which separate non-reimbursable medicines (with low aggregate scores) from those eligible for conditional reimbursement (with medium aggregated scores) or unconditional reimbursement (with high aggregate scores). This approach has been criticized for two major reasons: firstly, it reverts to the use of the same semi-rigid thresholds that it warns against within economic evaluation (e.g., ‘ICER-thresholds’) and secondly, it disregards the very different policy goals that different countries may follow when they decide about implementing some sort of HTA.

While the first argument is certainly valid, the underlying value concept is very different. In the case of ICER or cost/QALY thresholds, the underlying interpretation of value is built on one pivotal notion, cost-effectiveness, which is supposed to absorb and represent all relevant sources of value although in actual fact it faces clear limitations in doing so (patient convenience, patient-specific variations or patient-relevant outcomes). In BAS, the thresholds are applied after several sources of value have already been explicitly assessed against multiple decision criteria. Moreover, it is recommended that cut-off points should always be established in each BAS system in a way that they reliably separate clusters of medicines. This constitutes a significant difference to the GDP-linked or minimum wage-linked thresholds used in ’economic evaluation countries’.

The second argument is more nuanced. The original BAS model failed to differentiate between two very divergent policy goals (continuous assessment of reimbursement submissions vs. prioritization of medicines waiting for public funding) simply because it did not anticipate the second one. The model was proposed as a decision support tool for systems which ‘process’ manufacturer-initiated submissions in a continuous way but it soon became clear that it could serve for the prioritization of long lists of medicines waiting for funding decisions in countries where these are cyclical (mostly linked to the political cycle). This second use became popularly known as ‘backlog reduction’.

The purported use of balanced assessment may shape the decision rules to be applied and, in this regard, a more differentiated and granular approach is indeed necessary. During policy discussions, the following alternative decision rules have emerged besides the original logic based on cut-off points:

- *TOP X medicines to be reimbursed*. In this variant, scoring is performed but the score attained by a medicine is not measured against any cut-off point. Instead, a league table of medicines is set up and a pre-determined number of highest-ranking products (TOP 5, TOP 10, etc.) are adapted into public funding at the end of each period *without regard to* budget impact. Separate league tables may be set up for different budgets (e.g., outpatient vs. hospital) although this certainly introduces a strong opportunistic incentive for manufacturers to apply for funding in budgets where actual league tables ae relatively ‘weaker’. In this model, it is the payer’s remit and responsibility to find and release the necessary funds corresponding to the aggregated net budget impact (adjusted through managed entry agreements) of the medicines to be reimbursed (e.g., through generic or biosimilar policies or extra investment); for this, the effective date of public funding can be deferred by some time compared to the listing decision itself. Ideally, the frequency with which reimbursement formularies are opened up is pre-defined and known to all stakeholders. This, however, is not a formal requirement for this variant to be actionable. The radical assumption in this model is that the payer is ready and able to find and release the necessary funds, i.e., it accepts positive budget impact.
- *TOP X medicines to be reimbursed until the available budget is exhausted*. The previous variant may not be politically acceptable in financially constrained environments where payers are not ready or able to proactively look for available funds or acknowledge extra investment into pharmaceuticals. Accordingly, they may only unlock reimbursement formularies when funds are available (e.g., provided by government decision), and when they do so, they will only adopt new technologies up to the extent of available funds. In this case, the league table effectively becomes a ‘waiting list of new medicines’. Without budget guarantees, this variant does not meet the elementary principles of predictability but it may be the only politically viable scenario in legislations where pharmaceutical listing decisions are a means of political rationing linked to the political cycle. Its obvious drawback is that a product with exceptional clinical value but correspondingly high net budget impact can theoretically block the system indefinitely. A solution to this problem may be anchoring public pharmaceutical expenditure to GDP growth or, for technical reasons related to the logic of state accounts, a proxy of GDP growth. In this case, economic growth creates the ‘headroom’ for new listing decisions. (Naturally, in the case of economic contraction or stagnation, this anchoring mechanism will not provide funding to new medicines unless there are internal inefficiencies in the reimbursement system and these are detected and properly addressed.)
- *Either of these models with a minimum threshold*. Both variants above can be combined with a minimum threshold. In this case only medicines reaching a pre-determined minimum score will be included in the league table.
- *Moving threshold rule*. This rule has been proposed as an interesting conceptual alternative by the EFPIA HTA group [4]. In this logic, there is no pre-defined threshold. Rather, the budget impact of the medicine is assessed separately from its other sources of value (whether economic or clinical) and the higher the budget impact of a medicine, the higher the medicine needs to perform on other value dimensions to be eligible for public funding. For this system to be workable, tiers of budget impact need to be defined.

In league table systems, it must be regulated how the system deals with medicines which newly appear in the league table before other medicines, previously included therein, could receive public funding. As long as the assessment criteria and the assessment process are unchanged, it is permissible as a general rule that newly arriving medicines overtake previously assessed medicines although there may be well-specified ethical considerations that override this general guidance. Whenever the assessment logic changes (either through new criteria or through new score allocation mechanisms), the possibility to overtake is rather questionable and not recommended. It is also essential in all league table systems to have institutional guarantees that decision-makers must not handpick certain medicines from the list, i.e., no lower-scoring product can be listed before higher-scoring products have been listed.

### Value dimensions

**Figure 2.**
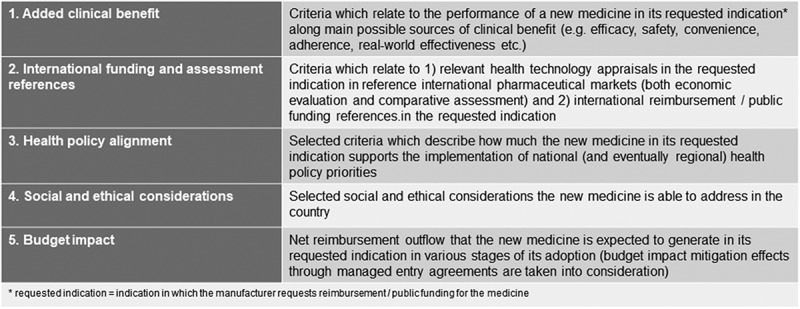
Proposed value dimensions in recent BAS applications.

Balanced assessment is organized around value dimensions within which different assessment criteria can be defined. The logic is similar to that of the balanced scorecard (BSC) in performance management [19]. The question is whether value dimensions can be pre-determined or each BAS application should define value dimensions *de novo*. Based on discussions around balanced assessment, there seems to be a preference for standard value dimensions but not necessarily those ones which were featured in the original model (Figure 2).

There seems to be consensus that *added clinical benefit* (in the original model: therapeutic value added), *social and ethical considerations* and *health policy alignment* are important value dimensions in the model. (The original model comprised social, ethical and policy alignment aspects as one dimension but this is rather a technical design question.) The role of economic dimensions (cost-effectiveness, budget impact and accessibility with funding in peer countries) has been much more vividly debated. This debate mainly revolves around three themes: (1) whether cost-effectiveness should be included as an assessment dimension or it does more harm than good, (2) related to this, whether referencing *any* external sources is an acceptable pragmatic shortcut or not, and (3) whether budget impact can be considered a separate assessment dimension and, as such, a source of product value.

The way balanced assessment incorporates cost-effectiveness analyses has proven to be quite controversial. The challenges of, and the limited rationale for, conducting localized cost-effectiveness analyses in the original target geographies for BAS was discussed in detail in the original article and has received widespread confirmation from various audiences. On the other hand, the BAS recommendation to substitute localized analyses through referencing of previous assessments in countries with more solid knowhow and methodological apparatus as ‘proxies’ has not been unanimously embraced due to the limited transferability of economic evaluations (which the original article anyhow acknowledged). The pragmatic claim of balanced assessment that the opportunity cost of building primary cost-effectiveness analyses on limited and low-quality data for decision-makers who have different information processing styles is likely to be higher than the implicit cost of transferring not fully transferable assessments has not been a sufficient response to critics. Thus, the debate has unfolded in different directions: for example, in its interpretation of BAS, the EFPIA HTA Group discarded cost-effectiveness analyses entirely. In a BAS proposal for Serbia, cost-effectiveness has been excluded, too, whereas cost-effectiveness has been retained in a similar initiative in Slovakia. Based on all these discussions, the authors of this article deem that there are institutional environments (e.g., with poor data availability, or shortage of experts) where it is little use imposing localized cost-effectiveness analyses on the value assessment system. Nevertheless, we recognize the need for explicitly addressing adoption feasibility when external indicators of cost-effectiveness are used.

The cost-effectiveness debate has expanded to a more general discussion about the use of any external references. The original BAS model included ‘*accessibility with public funding in peer countries*’ as an assessment dimension and it also used external referencing in comparative benefit assessment. While critics rightly say that accessibility with public funding is in no way any valid indicator of the value of the medicine, this was meant to be an auxiliary dimension on the economic side to ‘tranquillize’ governments concerned with losing control over listing decisions. Cynical as though it may seem, the overwhelming majority of payers (decision-makers) do consider this parameter, explicitly or implicitly, in their decisions and they will only accept any more balanced frameworks if this criterion is retained. Therefore, this can be interpreted as a ‘political’ dimension in the BAS model and the benefit of its incorporation is that it renders explicit some previously implicit payer considerations. In more recent models of BAS, external cost-effectiveness references, comparative benefit assessment references and funding references have been merged into one ‘external references’ dimension; this condenses external references into one auxiliary dimension and opens up the option of conducting localized appraisals of added therapeutic benefit. This, in turn, will require more local assessment competences and, preferably, well-embedded deliberative processes.

The question of budget impact was mentioned earlier but it also appears in a broader context. Some experts have asked if budget impact can be considered a value dimension. Others have pointed out that if the ‘*TOP X medicines to be reimbursed until the available budget is exhausted’* decision rule is used, budget impact should not qualify a medicine for any scores as this would be a sort of double-counting: it should then be treated as an external variable. There may not be conclusive answers to these questions. We believe that if a medicine releases funds for the health care system, it provides a valuable service, thus it is a source of value, albeit not of clinical value. This speaks for including budget impact as a value dimension or at least as one distinct economic criterion. Regarding the double-counting critique, we can accept it although a parallel interpretation can be that budget impact as a value dimension relates to the affordability of the particular medicine whereas budget impact as part of a decision rule reflects on the instantaneous general ability-to-fund of the health care system. Thus, the dual role of budget impact can be reconciled.

### Consistency issues

Some experts have pointed out that there may be other instances of double-counting in the original balanced assessment model. For example, efficacy and safety (which can be standard criteria in the ‘added clinical benefit’ dimension) are likely to be considered again through referencing to previous health technology assessments by international agencies. This criticism is certainly true: criteria in a BAS system may not be linearly independent. While this may introduce only minor multiplicator distortions into the assessment, it can always be managed through the elimination of external references – at the possible cost that more primary analyses will be needed which can be both expensive and time-consuming.

Another aspect that has been mentioned in discussions about pragmatic balanced assessment is the possible proliferation of fragmented and possibly interrelated criteria in the social and ethical dimensions as well as the risk that criteria serving vested interests are introduced into the system, for example during the consultation phase. Whereas the proliferation of social and ethical criteria with limited overall relevance is primarily the responsibility of system designers, the introduction of vested interests is a largely political issue. Romania’s scorecard-based quick HTA system [20] does not meet the requirements of BAS system for several reasons but the fact that its scorecard allocates 45 points out of a total of 145 to the availability of real-world evidence in Romania clearly exemplifies this phenomenon resulting in a scoring exercise which is very vaguely related to product value. Presumably the more experience is accumulated with balanced assessment models, the more easily such distortions can be avoided.

## Conclusions

The balanced assessment model has drawn attention to the importance of designing and implementing contextually sensitive (‘fit-for-purpose’) HTA architectures, and to the necessity of viewing health technology assessment as a pragmatic tool to inform decision-making rather than as scientific or research exercise only. The BAS model enables quick prioritization decisions in health care systems with significant ‘medicine backlogs’ and it also supports continuous (submission-driven) listing decisions and it minimizes the risk of ‘capture’ of the HTA system by particular interests.

Most methodological weak spots of the original model can be amended on the basis of the policy dialogue conducted since its inception. Some its shortcomings will remain as these are based on value choices. For example, when gauging the applicability of external references, policymakers must make trade-off decisions (e.g., lower costs, higher speed and less risk of system capture in exchange for less localization). Discussions have also revealed that a slightly different structure of value dimensions may be more convenient when used as standard dimension set: for this purpose, the authors recommend *added clinical benefit, international funding and assessment references, health policy alignment, social and ethical considerations and budget impact* (Figure 2). As discussed in depth in this article, cost-effectiveness can also be added as an assessment dimension but caveats apply. With these amendments, the robustness of the BAS model increases while its pragmatic focus is preserved.

## References

[1] Dankó D. Health technology assessment in middle-income countries: recommendations for a balanced assessment system. J Mark Access Health Policy. 2014;2(1):1–8. 10.3402/jmahp.v2.23181 PMC4865748 27226832

[2] Dankó D Proposal for a pragmatic value assessment framework for innovative medicines in Serbia. Presented at: ’How close are we to having up-to-date healthcare in Serbia?’ Policy Roundtable; 2016 4 8; Belgrade, Serbia.

[3] Dankó D, Petrova G Health technology assessment in the Balkans: opportunities for a balanced drug assessment system. Biotechnol Biotechnol Equip. 2015;28(6):1181–1189. 10.1080/13102818.2014.978636 PMC4433901 26019605

[4] EFPIA HTA Group represented by Bruce A Pragmatic decision-making framework. Presented at: HTA Policy Roundtable 2015 3 20; Vilnius, Lithuania.

[5] Gupta SK, Thomas D, Kumar Rai M Pharmacoeconomics & outcomes research guidelines for India. Lawrenceville (NJ): ISPOR HealthNet India; 2016 [cited 2017 4 11]. Available from: https://www.ispor.org/consortiums/asia/PEGuidelines_India_March2016.pdf

[6] Dankó D Opportunities for pragmatic health technology assessment in Gulf Cooperation Council (GCC) Member States. Presented at: Kuwait Conference on National Health Economics; 2015 5 11; Kuwait City, Kuwait.

[7] Marsh K Establishment of health technology assessment in russia: an interview with vitaly omelyanovsky. Bethesda (MD): Evidence Forum; 2015 [cited 2017 Febr 15]. Available from: http://www.evidera.com/newsletter/oct-2015

[8] Omelyanovsky VV, Fedyaeva VK, Rebrova OY Methodological recommendations for the application of multi-criteria analysis in health care [Методические рекомендации по применению многокритериального анализа в здравоохранении]. Moscow: FGBU CEKKMP Minzrdav Rossii; 2016 [cited 2017 4 11]. Available from: http://rosmedex.ru/wp-content/uploads/2016/12/MR-MCDA-23.12.2016.pdf

[9] Oortwijn W From theory to action: developments in value frameworks to inform the allocation of health care resources. Edmonton: HTAi Policy Forum; 2017 [cited 2017 4 25]. Available from: http://www.htai.org/index.php?eID=tx_nawsecuredl&u=0&g=0&t=1494651646&hash=cee7ed281487daaf36f332f416cfbe19ae457293&file=fileadmin/HTAi_Files/Policy_Forum/HTAi_Policy_Forum_2017_Background_Paper.pdf

[10] EUnetHTA HTA core model version 3.0. Diemen: EUnetHTA; 2016 [cited 2017 4 11]. Available from: http://eunethta.eu/sites/5026.fedimbo.belgium.be/files/HTACoreModel3.0.pdf

[11] Baltussen R, Jansen PM, Biljmakers L, et al Value assessment frameworks for HTA agencies: the organization of evidence-informed deliberative processes. Value Health. 2017 2;20(3):256–260. 28237205 10.1016/j.jval.2016.11.019

[12] Marsh K, IJzerman M, Thokala P, et al Multiple criteria decision analysis for health care decision making—emerging good practices: report 2 of the ISPOR MCDA emerging good practices task force. Value Health. 2016 Mar-Apr;19(2):125–137. 27021745 10.1016/j.jval.2015.12.016

[13] Drummond M, Barbieri M, Cook J, et al Transferability of economic evaluations across jurisdictions: ISPOR good research practices task force report. Value Health. 2009 6;12(4):409–418. 19900249 10.1111/j.1524-4733.2008.00489.x

[14] Mandrik O, Knies S, Kaló Z, et al Reviewing transferability in economic evaluations originating from Eastern Europe. Int J Technol Assess Health Care. 2016;31(6):434–441. 10.1017/S0266462315000677 PMC4824959 26961722

[15] EUnetHTA HTA core model for rapid effectiveness assessment of pharmaceuticals. Diemen, Netherlands: EUnetHTA; 2013 [cited 2017 4 11]. Available from: http://www.eunethta.eu/sites/default/files/sites/5026.fedimbo.belgium.be/files/Model%20for%20Rapid%20REA%20of%20pharmaceuticals_final_20130311_reduced.pdf

[16] Thokala P, Devlin N, Marsh K, et al Multiple criteria decision analysis for health care decision making–an introduction: report 1 of the ISPOR MCDA emerging good practices task force. Value Health. 2016 1;19(1):1–13. 26797229 10.1016/j.jval.2015.12.003

[17] Ontario Citizens Council The ontario citizens’ council report: towards a values framework. Toronto: OCC; 2011 [cited 2017 4 25]. Available from: http://www.health.gov.on.ca/en/public/programs/drugs/councils/report/report_values_framework.pdf

[18] HTAi Policy Forum HTA and value: assessing value, making value-based decisions, and sustaining innovation. Edmonton: HTAi Policy Forum; 2013 [cited 2017 4 25]. Available from: http://www.htai.org/index.php?eID=tx_nawsecuredl&u=0&g=0&t=1492010598&hash=47c7f08de331dc2532418a7c9cebfa94c1956b8c&file=fileadmin/HTAi_Files/Policy_Forum_Public/HTAi_Policy_Forum_Background_Paper_2013.pdf

[19] Kaplan RS, Norton DP The balanced scorecard: translating strategy into action. Watertown (MA): Harvard Business Review Press; 1996.

[20] Radu P, Chiriac ND, Pravat AM The development of the romanian scorecard HTA system. Value Health Reg Issues. 2016 9;10:41–47. 27881276 10.1016/j.vhri.2016.07.006

